# Eosinophils, Basophils, and Neutrophils in Bullous Pemphigoid

**DOI:** 10.3390/biom13071019

**Published:** 2023-06-21

**Authors:** Maren M. Limberg, Tobias Weihrauch, Natalie Gray, Nancy Ernst, Karin Hartmann, Ulrike Raap

**Affiliations:** 1 Division of Experimental Allergy and Immunodermatology, School of Medicine and Health Sciences, Carl von Ossietzky University Oldenburg, 26129 Oldenburg, Germany; 2 Division of Anatomy, School of Medicine and Health Sciences, Carl von Ossietzky University Oldenburg, 26129 Oldenburg, Germany; 3 Department of Dermatology, University of Lübeck, 23562 Lübeck, Germany; 4 Division of Allergy, Departments of Dermatology and Biomedicine, University Hospital Basel and University of Basel, 4031 Basel, Switzerland; 5 Research Center for Neurosensory Science, Carl von Ossietzky University Oldenburg, 26129 Oldenburg, Germany; 6 University Clinic of Dermatology and Allergy, University of Oldenburg, 26133 Oldenburg, Germany

**Keywords:** bullous pemphigoid, basophils, eosinophils, neutrophils, pruritus

## Abstract

Bullous pemphigoid (BP) is an autoimmune blistering skin disease, of which the incidence has increased in recent years. BP is characterized by circulating IgG and IgE autoantibodies against the hemidesmosomal proteins BP180 and BP230. Although autoantibodies trigger inflammatory cascades that lead to blister formation, effector cells and cell-mediated autoimmunity must also be considered as important factors in the pathogenesis of BP. The aim of this review is to outline the current knowledge on the role of eosinophils, basophils, and neutrophils in BP.

## 1. Introduction

Bullous pemphigoid (BP), which is the most common subepidermal autoimmune blistering disease [1], mainly affects the elderly and is associated with significant mortality and morbidity risks [2]. While the early phase of BP is clinically characterized by flares and sometimes wheals that are associated with a strong sensation of pruritus, flares in conjunction with tense, fluid-filled blisters are specific for the late stage of the disease [3]. Although BP is historically characterized by the production of IgG autoantibodies against the hemidesmosomal proteins BP180 and BP230 [4,5,6], studies in recent years have further elucidated the involvement of eosinophils, basophils, and neutrophils in the pathogenesis of BP. Now, infiltration of these immune cells into skin lesions is considered to be a predominant feature of BP. The objective of this review is to give an overview of the important role of eosinophils, basophils, neutrophils, and their granule products in the pathogenesis of BP.

## 2. Role of Eosinophils in the Pathogenesis of Bullous Pemphigoid

Eosinophilic granulocytes are bone marrow-derived cells of the myeloid lineage and were first described by Paul Ehrlich in 1879 [7]. The name was given due to the bright color when stained with eosin. Eosinophil granulocytes are primarily tissue-resident cells that act as key effector cells in inflammatory skin diseases and account for only 1–5% of circulating leukocytes in peripheral blood [8]. One of their functions is host defense, particularly in response to helminths and extracellular bacteria. Eosinophils bind and kill bacteria by generating extracellular DNA traps (EETs) that contain toxic granule proteins, such as the major basic protein (MBP), eosinophil-derived neurotoxin (EDN), and eosinophil cationic protein (ECP) [9,10]. 

**Figure 1:**
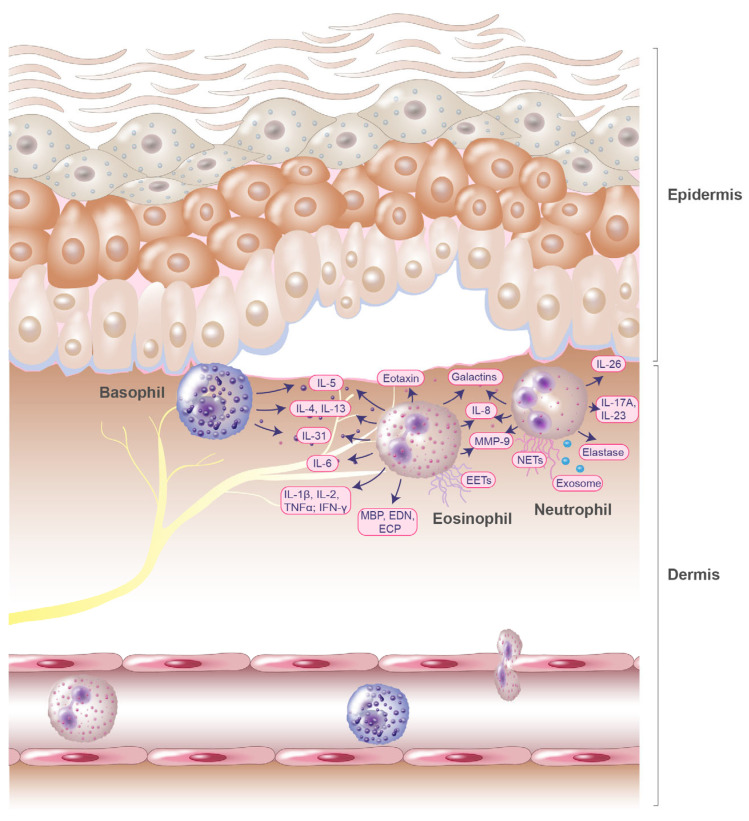
Potential role of eosinophils, basophils, and neutrophils in blister formation of bullous pemphigoid. Chemokine and cytokine gradients induce eosinophil, basophils, and neutrophil infiltration into the upper dermis. The inflammatory reaction is intensified by inflammatory mediators released from basophils, neutrophils, and eosinophils. Eosinophils release various cytokines, including the pruritic interleukin IL-31. Together with basophils, they secrete IL-4, IL-13, and IL-5. Additionally, eosinophils generate extracellular DNA traps (EETs) that contain toxic granule proteins, such as the major basic protein (MBP), eosinophil-derived neurotoxin (EDN), and eosinophil cationic protein (ECP). Eosinophils and neutrophils release IL-8, MMP-9, and galectins. Further, neutrophils release neutrophil extracellular traps (NETs) and exosomes.

The formation of a subepidermal cleft, accompanied by a significant eosinophilic infiltration, is a major histologic feature of BP [11,12]. Eosinophils have been shown to represent the prime early immune cells invading the skin in BP and are typically localized in the upper dermis of skin lesions in addition to lining the zone of the dermal–epidermal junction, but are also present in blisters and the peripheral blood of patients with BP [13]. Several studies demonstrate that eosinophils and their granule products (chemokines and cytokines) play a key role in the pathogenesis of BP [14,15,16] (Figure 1). Levels of various cytokines and chemokines, including interleukin (IL)-1β, IL-1α, IL-2, IL-4, IL-5, IL-6, IL-8, IL-15, IL-31, eotaxin, eosinophil colony-stimulating factor, tumor necrosis factor alpha (TNFα), and interferon-gamma (IFN-γ), are elevated in the sera and blister fluids of patients with BP [11,16,17,18,19,20,21]. Significant correlations have been determined for disease activity and blister fluid levels of IL-1β, IL-6, IL-8, TNF-α, and IL-5 (Table 1) [18,19].

BP blister fluids have been reported to contain not only products of eosinophils but also eosinophil chemotactic factors [37]. High eotaxin levels have been detected in BP blister fluid and were found to be significantly correlated with the number of dermal infiltrating eosinophils (Table 1) [21]. Immunohistochemical analysis conducted by Wakugava et al., demonstrated that eotaxin was strongly expressed in epidermal keratinocytes located around BP blisters [21], which have also been observed in other Th2-associated inflammatory skin diseases, such as atopic dermatitis [38]. These common expression patterns have been suggested to arise from eosinophils, which induce eotaxin-1 expression in keratinocytes through the release of ECP and EDN [39]. Moreover, blister fluids were found to contain high amounts of Th2 cytokines such as IL-5 [11,21]. IL-5 levels in serum exceeded those measured in blister fluids and were significantly increased in patients with BP (Table 1). Since IL-5 and eotaxin play a major role in eosinophil recruitment and function [40], this may partly explain the massive infiltration of eosinophils observed in BP (Figure 1). 

Pruritus is a major symptom in BP [41,42], suggesting a possible involvement of the pruritogenic cytokine IL-31. This was confirmed by Salz et al., by observing increased IL-31 concentrations in BP blister fluids (Table 1) [22]. The correlation of IL-31 levels with eosinophil numbers indicates that eosinophils might influence the regulation of IL-31 and itch in BP. More recently, our group was able to confirm these findings and additionally show that eosinophils are the major cellular source of IL-31 in BP [23]. These data further strengthen the hypothesis that eosinophils greatly contribute to itch in patients with BP via IL-31. IL-31 is produced not only by eosinophils but also by basophils [31], which are important players in the pathogenesis of BP, especially in the context of pruritus, which will be addressed later in this review.

Serum concentrations of eosinophil secretory granule products, such as MBP, EDN, and ECP, are also significantly elevated in patients with BP (Table 1) [22,24,25,26]. This suggests that the eosinophils of BP patients are in an activated state, as eosinophils release the content of their secretory granules upon degranulation. Findings from a study by Tsuda et al., support this assumption, as increased numbers of activated eosinophils were observed in blood samples from patients with BP [43]. Hypodense (activated) eosinophils were also found to be located in the basement membrane zone of skin samples, and direct adherence of eosinophils to basal keratinocytes was observed at blistering lesion sites [43]. By releasing granule proteins, eosinophils may directly damage basal keratinocytes, leading to dermo-epidermal separation. Furthermore, ECP and EDN have been reported to induce the expression of cytokines and chemokines such as IL-5, eotaxin-1, RANTES, and MMP-9 in keratinocytes. Both granule proteins initiate the release of reactive oxygen species (ROS) from keratinocytes and trigger their apoptosis [39]. In line with these findings, we showed that eosinophils in peripheral blood, skin, and blister fluids exhibited strong activation patterns, which were determined through measurement of CD69 expression [17]. Apoptosis in cultivated BP eosinophils was found to be increased, and in vivo initiation of apoptosis by caspase-3-positive eosinophils was confirmed in lesional BP skin sections [17], assuming that activated eosinophils have a shorter life span due to the release of inflammatory mediators. 

Additionally to releasing their secretory granules, eosinophils have also been observed to release EETs together with ECP in BP lesions (Table 1) [27]. Extracellular traps expand to up to 15 times the size of eosinophils and consist of network-like structures that contain DNA in conjunction with eosinophil granule proteins. Simon et al., analyzed 25 different eosinophilic skin diseases and detected extracellular DNA in skin samples from infectious and non-infectious inflammatory skin diseases. Interestingly, EETs were also found in skin biopsies of BP patients [27]. 

The proteome of BP blister fluids was characterized in a recent study, and 339 different proteins were identified, of which the eosinophil-derived proteins, MBP, eosinophil peroxidase, galectin-10, and the IgEε heavy constant region were unique and consistently associated with tissue eosinophilia (Table 1) [28]. The presence of relatively high levels of galectin-10 is particularly notable. Galectin-10, also known as Charcot-Leyden crystal protein (CLC-P), is a common protein of human eosinophils and a hallmark of active eosinophilic inflammation and proliferation [44]. However, galectin-10 is also produced by basophils [45], and the presence of galactin-10 in blister fluid may also be an indicator of basophil involvement in the disease process. 

In addition to galectin-10, the immunomodulator galectin-9 is also elevated in serum and in lesional skin samples of patients with bullous pemphigoid [29]. This is very interesting since galectin-9 is a potent chemoattractant for eosinophils, and massive recruitment of eosinophils to the skin is a feature of BP (Table 1). Galectin-9 expression has been detected in eosinophils, neutrophils, and keratinocytes [29].

Matrix metalloproteinases (MMPs) are increased in BP skin lesions [46] and may be involved in the mechanism of blister formation in BP too. This is of interest since eosinophils release MMP-9 upon stimulation with TNF [47] and metalloproteinase activity has been detected in BP blister fluids (Table 1) [48]. The extent of eosinophil involvement in the process of dermal epidermal separation through the release of MMPs was shown in a study by Stahle-Bäckdahl et al. [30]. They demonstrated that the abundant component of BP blister fluid, MMP-9 (92 kD gelatinase), is produced by eosinophils and that MMP-9 degrades the collagenous domain of a recombinant form of the 180 kD bullous pemphigoid autoantigen in vitro. These results suggest that the production and release of MMP-9 by eosinophils contribute significantly to blister formation in BP [30]. An in vitro study by Okada et al., investigated the role of MMP-9 in eosinophil basement membrane migration in Matrigel-coated chemotaxis chambers [49]. Eosinophil transmigration and degradation of the Matrigel layer were induced by platelet-activating factor (PAF), interleukin-5 (IL-5), and MMP-9, identified as key proteases. Notably, the substrate-degrading activity of MMP-9 was increased only in the presence of IL-5 and PAF [49]. These data indicate that MMP-9 release and activation mechanisms are involved in basement membrane transmigration of eosinophils.

In an ex vivo skin model, eosinophils previously activated with IL-5 were shown to induce dermal–epidermal junction separation in the presence of BP autoantibodies (Table 1) [50]. Moreover, eosinophil-mediated blister formation was found to be dependent on adhesion and Fcγ receptor activation. Blister formation also required elevated levels of ROS production [44], eosinophil degranulation, and EET formation [50].

In a study by Ständer et al., it was observed that an eosinophil-predominant inflammatory infiltrate was more frequently observed in patients that presented with intense subepidermal splitting [51]. This further underlies the role of eosinophils in BP. In recent years, the number of studies presenting data that supports the pathogenic role of IgE autoantibodies in BP, as well as their relationship with eosinophils, has increased. A mouse model that studied humanized anti-BP180-NC16A IgE and the high-affinity IgE receptor (FcεRI) showed that IgE autoantibodies induce eosinophil infiltration followed by blister formation in a dose-dependent manner. The eosinophil infiltration was further correlated with higher doses of anti-BP180-NC16A IgE [52]. Moreover, a high density of eosinophils in lesional BP skin inflammatory infiltrates was associated with increased seropositivity of anti-BP180-NC16A IgG [51]. In non-bullous pemphigoid, peripheral eosinophilia was also found to be significantly associated with elevated total IgE levels [53]. 

Although various observations support the pathogenic role of eosinophils in BP, few studies have examined the association between the number of tissue eosinophils, disease severity, and response to treatment. In a retrospective cohort study that included 233 patients with BP, a significant correlation was reported between peripheral blood and cutaneous eosinophil numbers and disease severity, as well as treatment outcomes [54]. The authors conclude that eosinophils may be used as a marker to predict disease severity in patients with BP [54]. The association between the amount of eosinophils in skin biopsy samples of 137 patients with BP and their demographic characteristics, comorbidities, disease severity, and treatment response was also evaluated in a very recently published retrospective study by Baum et al. [55]. Contrarily, they found no correlations between eosinophil numbers and disease severity, but their quantity may serve as a prognostic factor for treatment outcome. Recent studies, however, show that the inflammatory early phase of BP is associated with an infiltration of basophils that correlates with eosinophils, indicating a role for both granulocytes in blister development and early inflammation [13].

## 3. Role of Basophils in the Pathogenesis of Bullous Pemphigoid

Basophils have also been detected by Paul Ehrlich and are relatively rare cells, that represent less than 1% of peripheral blood leukocytes in humans [56]. They are characterized by a constitutive expression of FcεRI [57,58]. Basophils are important early producers of type 2 mediators such as IL-4 and IL-13, which drive inflammation and pruritus [59,60,61,62]. The predominance of the cytokines IL-4, IL-5, and IL-13 that can be detected in biopsies of lesional skin from patients with BP (Table 1) [20] suggests that Th2-type immunity is involved in BP pathogenesis. Beside eosinophils, basophils are key effector cells of inflammatory skin diseases. Recent studies reveal that basophils play a more important role in inflammatory skin diseases than previously thought. Infiltration of basophils into the skin has been observed in several inflammatory skin disorders, including atopic dermatitis (AD), prurigo, and urticaria, and, interestingly, also in autoimmune skin diseases such as BP [63]. 

While the accumulation of eosinophils in the skin tissue is a common finding in BP [16], basophils also infiltrate BP skin lesions and are involved in the induction of Th2 immunity alongside eosinophils (Figure 1). The involvement of basophils in the development of IgE-mediated chronic allergic skin inflammation and increased serum IgE levels in patients with BP [64] further suggest a pivotal role for basophils in BP. However, there are very few studies investigating the role of basophils in the pathogenesis of BP.

Infiltration of basophils into lesional BP skin was first detected in a study by Dvorak et al., in 1982 [65], where the inflammatory response in lesions of a BP patient was evaluated at different stages of disease progression by electron and light microscopy. Infiltration of immune cells into lesions was initially described for lymphocytes and later for eosinophils and basophils. Light microscopy showed basophils mainly in clinically prominent lesions and adjacent normal skin. Intralesional eosinophil location was associated with proximity to degranulated basophils. 

The extent of basophil infiltration into the skin in other inflammatory skin diseases was investigated in a study by Ito et al. [63]. Basophil infiltration was analyzed in 136 samples from 24 skin diseases through immunohistochemical staining. Contrary to previous assumptions, basophils were detectable in lesions of many diseases and conditions such as AD, prurigo, urticaria, drug eruptions, eosinophilic pustular folliculitis, insect bites, scabies, Henoch-Schönlein purpura, and dermatomyositis, as well as in BP. Interestingly, basophil infiltration was observed in nine of ten BP skin samples, a frequency that exceeds that seen in AD. Additionally, CD203c expression, a marker for blood basophil activation, was investigated by flow cytometry and found to be increased in patients with BP when compared to healthy donors [63]. 

The pathophysiological mechanisms of itch were investigated in BP patients in a recent study by Hashimoto et al. [66]. Lesions from 24 patients with BP were analyzed through immunofluorescence staining. The number of dermal infiltrating basophils was found to be significantly increased in BP skin sections compared to healthy controls. Interestingly, the number of dermal basophils significantly correlated with the severity of pruritus, highlighting the involvement of basophils in BP-associated itch. Beside basophils, eosinophils, SP, NK1R, IL-31, IL-31RA, OSMR, IL-13, and periostin were also correlated with the severity of pruritus, and eosinophils were identified as a major source of IL-31 [66]. 

The dual role of basophils in the development and resolution of BP was confirmed in a recent study by Kimura et al. [13], where erythematous and bullous lesions from a total of 25 patients with BP were compared by histopathology, immunohistochemistry, and electron microscopy. The number of basophils in the early phase of BP was found to be positively correlated with the number of eosinophils. Interestingly, basophil numbers were significantly higher in the bullous phase of BP. Together, the findings of this study suggest that basophils are involved in the development and resolution of BP, in part via cell-cell contacts with eosinophils or M2 macrophages [13].

## 4. Role of Neutrophils in the Pathogenesis of Bullous Pemphigoid

Neutrophils, which were first described by Max Johann Sigismund Schultze in 1865 [67], are abundant cells that comprise 50–70% of all circulating leukocytes [68]. Functioning as first responders, neutrophils play a role in host defense against pathogens such as bacteria, fungi, and protozoa. The nucleus of mature neutrophils is segmented, and the cytoplasm is enriched with granules and secretory vesicles. Like all other granulocytes, neutrophils develop in the bone marrow from committed myeloid progenitor cells [68].

The correlation that has been observed between neutrophil counts and disease severity in BP indicates a role for these leukocytes in the pathogenesis of BP [69]. The first evidence that neutrophils play an essential role in subepidermal blister formation arose from observations in an experimental BP model [70]. BALB/c mice treated with specific neutrophil-removing antibodies exhibited no evidence of inflammatory infiltration or blister formation after anti-mBP180 IgG application [70].

It has been reported that in patients with BP, the C-X-C motif chemokine ligand 10 (CXCL10) induces the secretion of MMP-9 from neutrophils (Table 1). This release was greater than that observed in healthy control subjects [35]. Nevertheless, eosinophils appear to be the main source of MMP-9 release at the site of blister formation in BP lesions, as gelatinase (MMP-9) mRNA expression has only been detected in eosinophils and not in other immune cells such as neutrophils [30]. Although neutrophils contain immunoreactive 92-kD gelatinase, active expression occurs exclusively in eosinophils [30]. Besides MMP-9, neutrophils also release elastase. Both compounds have been shown to cleave the BP180 antigen into tripeptides, such as proline–glycine–proline (PGP) (Table 1). PGP, in turn, functions as a matrikine that potentially acts as a chemoattractant for neutrophils. Together with the enhanced release of elastase from neutrophils, PGP contributes to the amplification of the inflammation loop [34] (Figure 1). In a study by Verraes et al., the contribution of neutrophil elastase (NE) to the degradation of BP180 autoantigens and to subepidermal blistering in BP was demonstrated [71]. The same effect could not be observed for MMP-9. Additionally, neutrophil elastase and MMP-9 were observed to be present in skin biopsies of bullous pemphigoid lesions [71]. In blister fluids, only NE could be detected in the active enzyme form. In contrast, MMP-9 in blister fluid is only present as a proenzyme [71]. This important role of the NE in antibody-induced blister formation was confirmed in vitro in an experimental BP model [72]. Furthermore, in vivo application of NE to murine skin induced BP-like epidermal–dermal separation [72]. 

A knockout experiment in mice revealed that, in addition to NE, neutrophil-related Fcγ receptors (analogons to human FcγIIA and FcγIIIA) are crucial for antibody-induced dermal–epidermal separation [73]. This observation underlines the suggested role of neutrophils in blister formation. Neutrophils are also capable of releasing neutrophil extracellular traps (NETs) in a process called NETosis by undergoing apoptosis. NETosis can also occur in a non-lytic form. NETs consist of cytosolic and granule proteins, which are assembled on a scaffold of decondensed chromatin. Their immunological function is to trap and kill microorganisms [74]. However, enhanced formation of NETs has also been found to be associated with the presence of autoantibodies in autoimmune diseases [75]. In a recent study, higher amounts of NETs were observed in skin lesions, serum, and blister fluids of BP patients than in corresponding samples of healthy control subjects (Table 1) [36]. The NETs in skin lesions were found to be located in the papillary dermis, on the margin of the dermal–epidermal separation [32]. Furthermore, the amount of NETs, which were assessed by levels of myeloperoxidase–DNA complexes in serum and blister fluids, was observed to be positively correlated with anti-BP180-NC16A antibodies, which function as markers for disease activity [36]. It was further shown that neutrophils from BP patients produce more NETs under resting conditions than those of healthy patients. Additionally, serum and blister fluids from BP patients enhanced NETosis not only in BP neutrophils but also when applied to those of healthy subjects [36]. In contrast, the serum of healthy donors did not upregulate NETosis in neutrophils. This indicates that inflammatory mediators present in BP cause neutrophils to be more susceptible to NETosis [36]. The cytokines IL-17A and IL-23 were reported to be elevated in the serum of BP patients who were at risk of disease recurrence. Stimulation of neutrophils with BP sera that contained IL-23 or IL-17A showed that these cytokines enhanced the release of NETs (Table 1) [32]. This is in line with the findings that in vitro, neutrophil activation occurs as a result of IL-17A stimulation [76]. IL-17A is further known to induce the expression of MMP-9 and neutrophil elastase [77]. Furthermore, dermal–epidermal separation through anti-BP180 IgG is inhibited by anti-IL17A treatment [76]. However, IL-23 was shown to have increased NET-inducing qualities compared to IL-17A, which has even been observed to reduce the NET-releasing action of IL-23 when both interleukins are added to BP sera in combination. The corticosteroids methylprednisolone and compound A have also been observed to inhibit the formation of NETs [32]. Further, BP180–NC16A immune complexes have been found to induce increased NET formation in neutrophils from healthy donors and BP patients. Concentrations of the Fcγ receptor, nicotinamide adenine dinucleotide phosphate (NADPH), and protein-arginine deiminase type 4 (PAD4) influence NET formation in a dose-dependent manner [36]. Furthermore, NETs have been observed to contribute to the differentiation of B cells into plasma cells via activation of the p38 mitogen-activated protein kinase (MAPK) cascade. Stimulation with NETS isolated from BP patients increases BP180-NC16A antibody titers in the supernatants of BP B cells but not in the B cell supernatants of healthy subjects [36]. 

Previously, Margaroli et al. investigated the release of mediators and compartmentalization of immune cells in BP [78]. Immunohistochemical analysis of BP skin sections demonstrated that eosinophils were the predominant immune cells in the skin and that neutrophils were present in lower amounts. A different pattern was observed in BP blister fluid. Here, neutrophils were the most prevalent cells. Blister fluid was also found to contain monocytes/macrophages and T cells, but very few eosinophils and B cells [78]. Furthermore, surface expression of leukotriene A4 hydrolase and NE was increased in neutrophils, monocytes/macrophages, and eosinophils from blister fluids. Additionally, interleukins (IL)-1α, IL-1β, IL-8, IL-10, IL-18, monocyte colony-stimulating factor (M-CSF), and vascular endothelial growth factor (VEGF) were observed to be elevated in blister fluid [78]. In a retrospective study, leucocyte ratios of BP patients were calculated from data of hospital medical records [79]. The neutrophil-to-lymphocyte ratio was significantly increased in the BP group, while the neutrophil-to-eosinophil ratio was decreased. Thus, it was concluded that the neutrophil-to-lymphocyte ratio can be used as a diagnostic tool for subtyping autoimmune bullous disorders [79].

Extracellular DNA levels and increased levels of circulating interleukin-26 (IL-26) have been detected in patients with BP, though how extracellular DNA contributes to the pathogenesis of BP is still unclear. A previous study highlighted a possible mechanism by which IL-26–DNA complexes in the upper dermis and inside the blisters mediate the dermal–epidermal separation in BP (Table 1) [33]. This was investigated in a modified cryosection model of bullous pemphigoid using human tissue. IL-26–DNA complexes increased the secretion of inflammatory cytokines in monocytes and neutrophils. Additionally, the complexes induced the production and activity of proteases from co-cultured monocytes and neutrophils, which then triggered the splitting of BP180 in keratinocytes and dermal–epidermal separation [33]. Beside extracellular DNA, extracellular vesicles also play an important role in BP. These extracellular vesicles have been shown to carry tissue-specific autoantigens in autoimmune diseases [80]. In a recent pilot study, BP180 was detected in extracellular vesicles of blister fluid in three of six patients [81]. The proinflammatory role of exosomes, which are a subpopulation of extracellular vesicles, in blister fluids of BP patients was characterized by Fang et al. [34]. They observed that blister fluid-derived exosomes induce neutrophil recruitment through interleukin-8 (IL-8). The group also suggested that keratinocytes and skin-infiltrating granulocytes, such as neutrophils, are possible sources of exosomes in the blister fluid of BP patients [34]. IL-8 and IL-6 were also observed to be released from keratinocytes upon BP IgG binding to BP180 [82]. Blister fluids further contained higher levels of IL-8 than serum samples [83]. 

As mentioned before, Pruessmann et al., suggested that galectin-9 may play a role in the pathogenesis of bullous pemphigoid [29]. In addition to eosinophils, the expression of galectin-9 was also significantly increased in neutrophil granulocytes in skin lesions of BP patients (Table 1) [29]. Galectins are important checkpoints of the immune system. One case report described two cases in which neutrophils predominated in immune checkpoint inhibitor-induced BP [84].

A study by de Graauw et al., demonstrated that the synergistic effects of neutrophils and monocytes influence dermal–epidermal separation [85]. Human foreskin cryosections were incubated with BP serum and purified leukocytes from healthy donors. When sections were incubated with neutrophils and monocytes in combination, the separation of the skin layers was far more pronounced than when sections were incubated with either neutrophils or monocytes alone. It was further observed that the synergism was associated with increased production of ROS and enhanced release of MMP-9 from neutrophils. Moreover, the monocyte/neutrophil-induced dermal–epidermal separation was reported to be dependent on cell adhesion and Fcγ receptor binding [85]. The corticosteroid methylprednisolone was found to inhibit immune complex induced production of ROS from neutrophils and also reduce lipopolysaccharide- and immune complex-mediated degranulation. These effects explain the therapeutic efficacy of methylprednisolone [86]. 

In addition to monocytes, mast cells have been observed to affect neutrophils in an experimental model of bullous pemphigoid. The role of mast cells in neutrophil recruitment during subepidermal blister formation in BP was described in a study by Chen et al. [87]. Here, mast cell degranulation led to the infiltration of neutrophils into the skin and subsequent subepidermal blistering in wild-type mice that were treated with anti-mBP180 IgG antibodies [87]. In contrast, mice that were genetically deficient in mast cells or treated with mast cell degranulation inhibitors showed reduced numbers of infiltrating neutrophils at the injection site. This reduction in neutrophil counts can be counteracted by an intradermal injection of IL-8 [87]. 

To summarize, these findings indicate that neutrophils play a pivotal role in the pathogenesis of bullous pemphigoid by directly contributing to dermal–epidermal separation through the release of neutrophil elastase. The formation of NETs, which are associated with disease severity by promoting elevated antibody titers through B cell differentiation, further highlights the importance of neutrophils in the pathogenesis of BP.

In conclusion, eosinophils, basophils, neutrophils, and their granule products are important players in the pathogenesis of BP. Infiltration of these cells from the peripheral blood into the skin is a predominant feature of BP. Further studies are needed to obtain a deeper understanding of the role of eosinophils, basophils, and neutrophils in BP and to drive the development of new therapeutic approaches for this autoimmune blistering and itching skin disease.

## Figures and Tables

**Table 1:** Upregulation and functions of mediators from eosinophils, basophils, and neutrophils in the pathogenesis of BP.

Immune Cells	Mediators	Upregulation and Functions	Ref.
Eosinophils	IL-15, eosinophil colony-stimulating factor	Elevated in blister fluids of patients with BP	[16]
	IL-2, IL-4, IFN-γ	Increased serum levels in patients with BP	[19]
	IL-1β, IL-8, TNFα	Increased serum levels in patients with BP; significant correlations with disease intensity	[19]
	IL-6, IL-8	Increased serum levels in patients with BP	[18,19]
	Eotaxin	Upregulated in blister fluids; significantly correlated with the number of dermal infiltrating eosinophils	[21]
	IL-5	Elevated serum levels; significant correlations with disease intensity; increased amounts in blister fluids; induces dermal–epidermal separation upon stimulation of eosinophils	[11,16,18,19,20,21]
	IL-31	Increased concentrations in BP blister fluid; secreted by human eosinophils	[22,23]
	MBP, EDN, ECP	Significantly elevated in patients with BP	[22,24,25,26]
	EETs	Released in BP lesions	[27]
	Galectin-10	Found in proteome of BP blister fluids	[28]
	Eosinophil peroxidase	Found in proteome of BP blister fluids	[28]
	Galectin-9	Elevated in serum and in lesional skin; chemoattractant for eosinophils	[29]
	MMP-9	Abundant component blister fluid MMP-9 is produced by eosinophils; degrades BP180 autoantigen	[30]
Basophils	IL-4, IL-5, IL-13	Detected in biopsies of lesional skin from patients with BP	[20]
	IL-31	Increased concentrations in BP blister fluids; released by human basophils	[22,31]
Neutrophils	IL-17A, IL-23	Elevated in serum of BP patients; enhance the release of NETs; IL-17A induces expression of MMP-9 and neutrophil elastase	[32]
	IL-26, extracellular DNA	IL26–DNA complexes mediate dermal–epidermal separation and increase secretion of inflammatory mediators from neutrophils	[33]
	IL-8	Derived from blister fluid exosomes; induces neutrophil recruitment	[34]
	MMP-9	Increased induction by chemokine CXCL10 in BP	[35]
	Neutrophil elastase	Cleaves BP180 antigen into tripeptides	[34]
	NETs	Higher amounts in skin lesions, serum, and blister fluids in BP	[36]
	Galectin-9	Increased in neutrophils in BP skin lesions	[29]

## Data Availability

Not applicable.
